# Apancreatic pigs cloned using Pdx1-disrupted fibroblasts created via TALEN-mediated mutagenesis

**DOI:** 10.18632/oncotarget.23301

**Published:** 2017-12-14

**Authors:** Jin-Dan Kang, Hyojin Kim, Long Jin, Qing Guo, Cheng-Du Cui, Wen-Xue Li, Seokjoong Kim, Jin-Soo Kim, Xi-Jun Yin

**Affiliations:** ^1^ Jilin Provincial Key Laboratory of Transgenic Animal and Embryo Engineering and Department of Animal Science, Yanbian University, Yanji 133002, China; ^2^ Center for Genome Engineering, Institute for Basic Science, Gwanak-gu, Seoul 151-747, South Korea; ^3^ ToolGen, Inc., Byucksan Kyoungin Digital Valley 2-Cha, Geumcheon-Gu, Seoul 153-023, South Korea; ^4^ Present/Current address: Department of Biosystems Science and Engineering, ETH Zurich, Basel CH-4058, Switzerland

**Keywords:** PDX1, TALEN, SCNT, pancreas, pig

## Abstract

Pancreatic and duodenal homeobox 1 (PDX1) plays a crucial role in pancreas development, β-cell differentiation, and maintenance of mature β-cell function. In this study, we designed a strategy to produce PDX1-knockout (KO) pigs. A transcription activator-like effector nuclease (TALEN) pair targeting exon 1 of the swine PDX1 gene was constructed. Porcine fetal fibroblasts (PFFs) were transfected with the TALEN plasmids plus a surrogate reporter plasmid. PDX1-mutated PFFs were enriched by magnetic separation and used to produce homozygous PDX1-KO pigs via a two-step somatic cell nuclear transfer (SCNT) cloning process. In the first SCNT step, we obtained eight fetuses, established PFF cell lines, and analyzed PDX1 gene mutations by T7 endonuclease 1 assays and Sanger sequencing. Five fetuses showed mutations at the PDX1 loci with two biallelic mutations and three monoallelic mutations (mutation rate of 62.5%). In the second step, a PDX1 biallelic mutant PFF cell line with a 2 bp deletion in one allele and a 4 bp insertion in the other allele was used as a donor to generate cloned pigs via SCNT. From 462 cloned embryos transferred into two surrogates, nine live piglets were delivered. These piglets at birth were not clearly distinguishable phenotypically from wild-type piglets, but soon developed severe diarrhea and vomiting and all died within 2 days after birth. Dissection of PDX1-KO piglets revealed that the liver, gallbladder, spleen, stomach, common bile duct, and other viscera were present and normal, but the pancreas was absent in all cases.

## INTRODUCTION

Pancreatic and duodenal homeobox 1 (PDX1), a transcription factor identified a decade ago during a search for insulin transcription regulatory mechanisms, plays a critical role in an array of pancreatic and islet functions [1]. The critical role of PDX1 in pancreatic development was first demonstrated in the Pdx1-knockout (KO) mouse model (Pdx1^−/−^) in which pancreatic agenesis is observed, coupled with a failure of the pancreatic bud to grow [2]. Naturally occurring non-functional mutations in the PDX1 gene have been reported in people born with pancreatic agenesis [3–6]. One pancreatic agenesis patient initially presented with hyperglycemia. Despite aggressive dietary and insulin therapy, she failed to gain weight in the first 18 days of life. She was homozygous for a point deletion in the PDX1 gene. The patient received insulin and pancreatic enzyme replacement therapy and developed normally [4].

Considering the closer similarity of humans with pigs than with mice in terms of anatomy, physiology, and the genome, pigs can serve as a large animal model to study human metabolism and physiology [7, 8]. Given that various mutations in the human PDX1 gene give rise to a spectrum of disease phenotypes, ranging from the complete absence of the pancreas to maturity onset diabetes of the young [4, 9], pigs with PDX1 gene mutations would be a useful animal model to study the association of diabetes mellitus and human pancreatic agenesis diseases. Transcription activator-like effector nuclease (TALEN)-mediated genome modification has been successfully applied to generate animals with monoallelic or biallelic gene modifications, such as mice [10], pigs [11], and even monkeys [12]. Therefore, the objective of this study was to produce PDX1^−/−^ pigs using a combination of TALEN technology and somatic cell nuclear transfer (SCNT).

We propose that these animals are useful resources to study human pancreatic agenesis and to develop novel drugs such as pancreatic enzyme replacement therapies. In addition, PDX1-KO pig cells and human induced pluripotent stem cells can be used to generate human pancreases in pigs via blastocyst complementation, which will open up new avenues for organ transplantation.

## RESULTS

### Validation of PDX1-targeting TALEN activity and TALEN-driven genome editing

**Figure 1:**
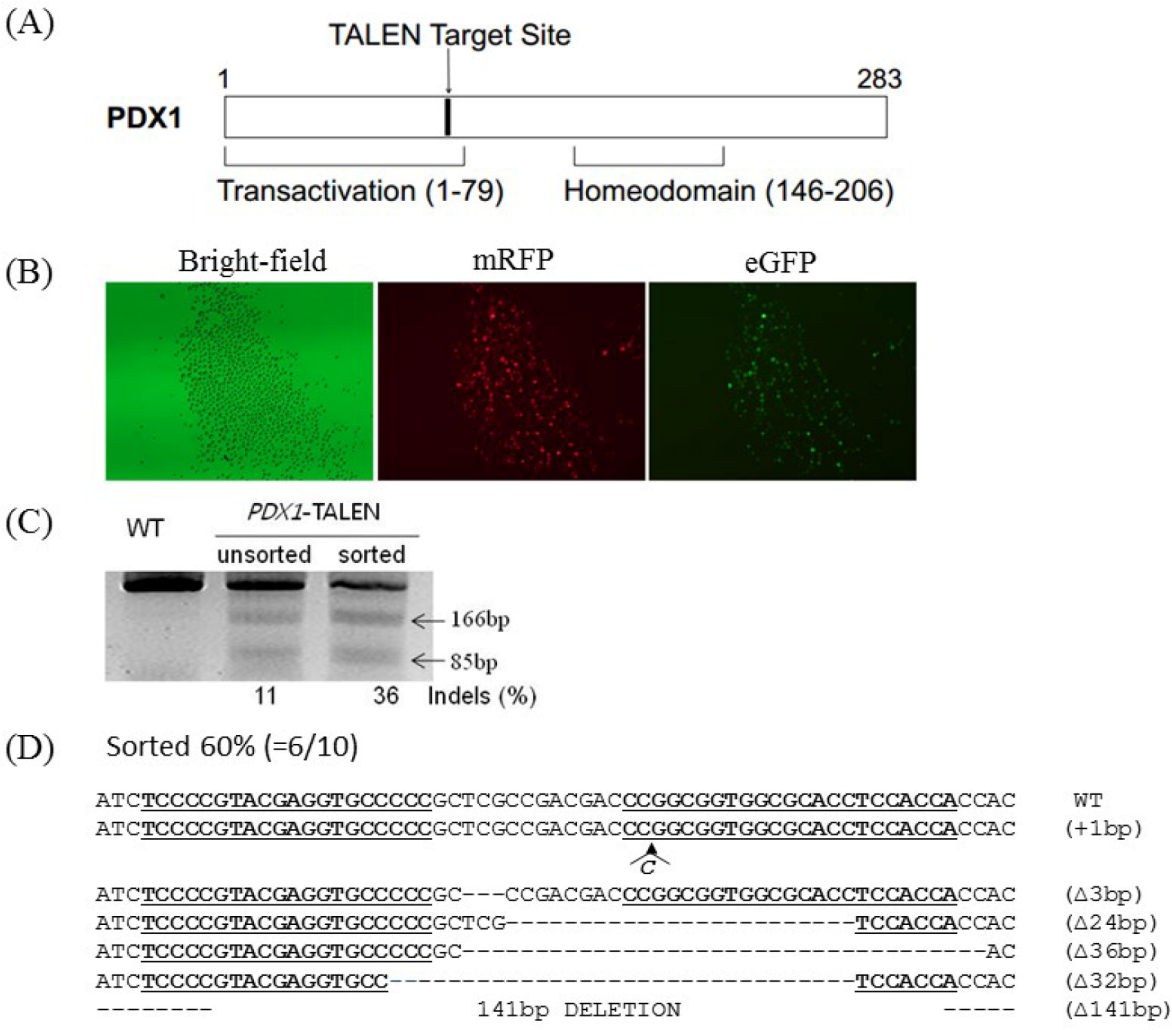
Generation of *PDX1*-disrupted PFFs using *PDX1*-TALEN **(A)** Schematic representation of functional domains of *PDX1* and the *PDX1*-TALEN target site. **(B)** Magnetic separation-mediated enrichment of *PDX1*-disrupted PFFs. PFFs were cotransfected with *PDX1*-targeting TALENs and reporter plasmids, followed by magnetic separation of H-2K^k^-expressing cells designed to express both mRFP and eGFP. Fluorescence microscopy examination was performed 1 day after magnetic separation. Magnification, 40×. *PDX1*-TALEN-driven mutations at endogenous chromosomal sites in magnetic separated PFFs. **(C)** The T7E1 assay were used to detect *PDX1*-TALEN-driven mutations. Arrows indicate the expected positions of DNA bands cleaved by T7E1. Mutation frequencies (Indels (%)) were calculated by measuring the band intensities). **(D)** DNA sequences of the *PDX1* wild-type (WT) and mutant clones. The region of the target sequence of the *PDX1*-TALEN is shown in underlined, deleted bases are indicated by dashes. The column on the right indicates the number of inserted or deleted bases.

We designed a TALEN pair tartgeting to transactivation domain in exon 1 of the swine PDX1 gene (Figure 1A). The activity of the PDX1-TALEN was evaluated using a surrogate reporter system. In these assays, the induction of small indel mutations in the TALEN target sequence upstream of the inactive eGFP and H-2K^k^ cell surface reporter genes can restore the expression of these genes, while mRFP is constitutively expressed independent of TALEN activity. Primary porcine fetal fibroblasts were transformed with PDX1-targeting TALEN and surrogate reporter plasmids. Cells expressing eGFP were detected only upon co-transfection of TALEN and reporter plasmids, validating the activity of the PDX1-TALEN in primary porcine fetal fibroblasts. Incubation of transfected cells with magnetic beads (MACSelect K^k^ microbeads; Miltenyi Biotech, Germany) conjugated to an anti-H-2K^k^ antibody and elution through a MACS LS column (Miltenyi Biotech) led to the separation of H-2K^k^-expressing cells [13, 14]. These H-2K^k^-positive cells were enriched with eGFP-expressing cells (Figure 1B).

Additionally, to directly evaluate the extent of TALEN-induced mutations, genomic DNA isolated from sorted and unsorted cell populations was subjected to T7E1 assays and Sanger sequencing. PCR amplified a 251 bp band, whereas PCR products from cells transformed with the PDX1-TALEN showed a cleaved band upon T7E1 digestion, confirming the presence of mutations in the transformed cells. The PDX1 mutation frequency was higher in magnetically sorted cells than in unsorted cells (36% vs. 11%, Figure 1C). 6 out of 10 clones harbored mutations at the PDX1-TALEN-tarted site (60%) and two of them harbored 141bp large deletion in PDX1 gene (Figure 1D).

### Production of PDX1-KO fetuses by SCNT

**Figure 2:**
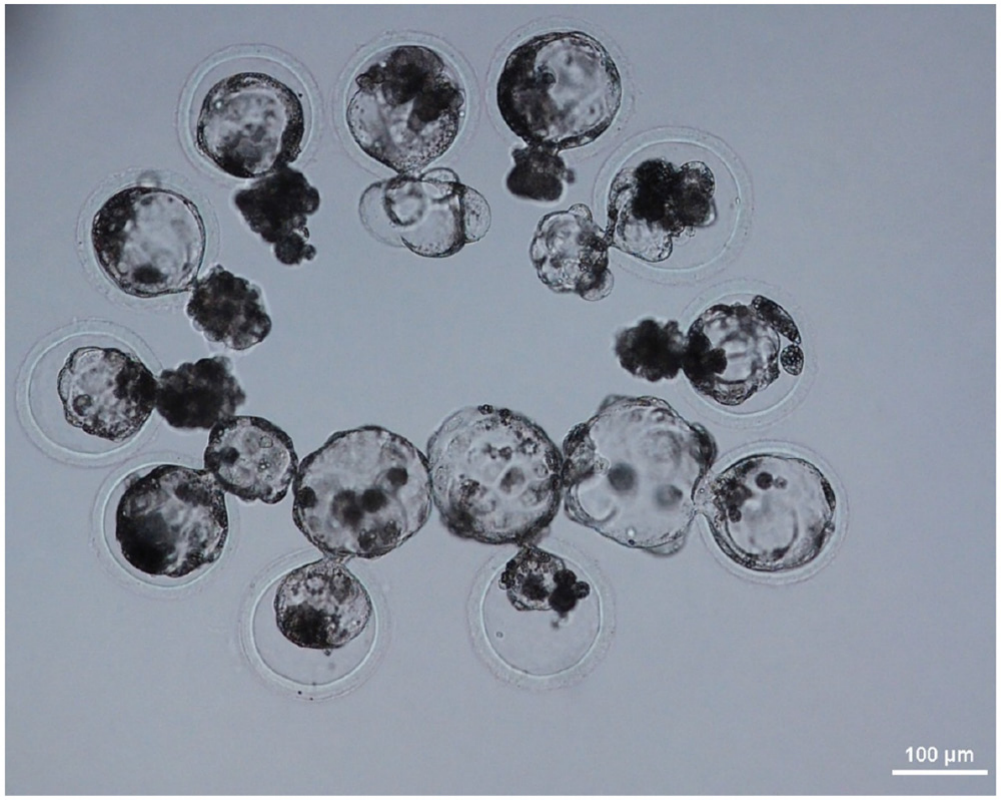
Day 7 SCNT blastocysts derived from PFFs transfected with PDX1-TALEN plasmids

**Figure 3:**
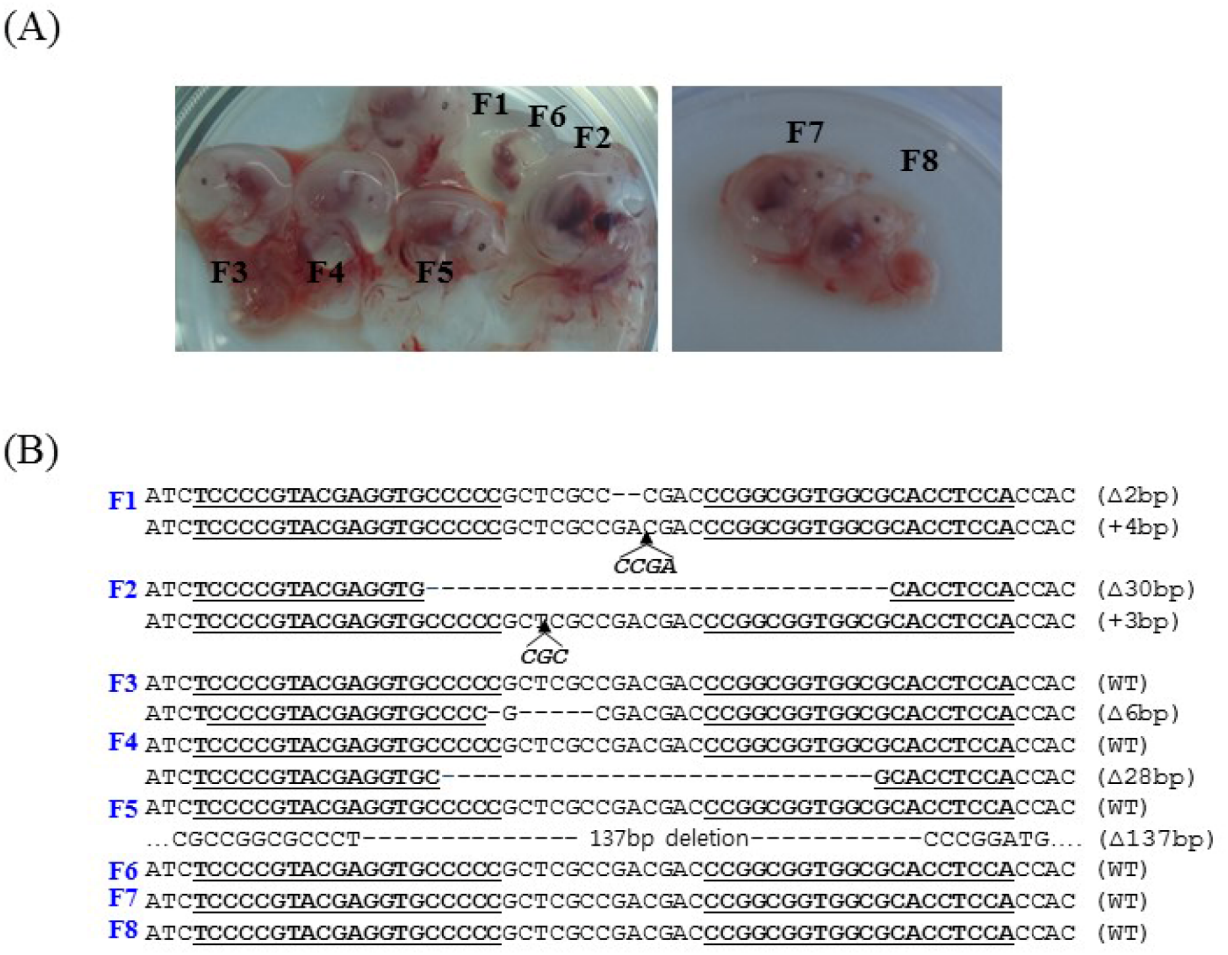
Photographs of the fetuses and *PDX1* sequences **(A)** Fetuses derived from TALEN-treated PFFs at day 26 (left) and day 29 (right). **(B)** *PDX1* sequences from TALEN-treated fetuses. DNA sequences of the WT and mutant clones, with TALEN recognition sites underlined, deleted bases indicated by dashes, and inserted bases in italics. Two of eight fetuses harbored biallelic mutations and three harbored monoallelic mutations in *PDX1*.

PFFs collected from females were transfected with the PDX1-TALENs and reporter plasmids by electroporation, and transfected cells were enriched magnetically as described previously [11]. H-2K^k^-positive cells expressing both mRFP and eGFP (Figure 1B) were cultured for two additional days and were used as donor cells in SCNT. The competency of reconstructed embryos cultured *in vitro* was investigated by examining the development of blastocysts. The blastocyst development rate (18.1% vs. 19.5%, Figure 2) and the mean number of cells per blastocyst (38.7 ± 8.1 vs. 39.7 ± 4.2) were similar in embryos reconstructed with transformed and non-transformed donor cells (Table 1). To produce PDX1-mutant fetuses, 386 embryos cloned using H-2K^k^-positive PFFs were transferred into two surrogate mothers (R725 and R801). Eight fetuses were surgically collected from R-725 after 26 days of gestation, and two fetuses (Figure 3A) were surgically collected from R-801 after 29 days of gestation (Table 2).

**Table 1:** *In vitro* development of reconstructed pig embryos with donor PFFs transfected with or without *PDX1*-TALEN plasmids

Donor cell type	No. ofembryos	No. of2–4-cell embryos (%)	No. ofblastocysts (%)	No. of cells per blastocyst (mean ± SEM)
Non-transfected PFF	232	204 (88.0)	42 (18.1)	38.7 ± 8.1
Transfected PFF	261	222 (85.1)	51 (19.5)	39.7 ± 4.2

**Table 2:** Summary of the production of *PDX1*-KO fetuses using *PDX1*-TALEN and SCNT

Recipient	No. of embryos transferred	No. offetuses	Days of gestation	Fetal fibroblast cell lines	Mono-allelic cell lines (%)	Bi-allelic cell lines (%)
R725	205	6	26	F1, F2, F3, F4, F5, F6	F3, F4, F5(50.0)	F1, F2(33.3)
R801	181	2	29	F7, F8	-(0%)	-(0%)

### Detection of mutations and genotyping of PDX1-mutant fetuses

Fibroblast cell lines from the eight fetuses were analyzed individually by T7E1 assays and by sequencing PCR products covering the target locus. Mutations at the target locus (PDX1 gene) were present in five of the eight fetuses (62.5%; Table 2). Two fetuses (F1 and F2) carried biallelic mutations and three fetuses (F3, F4, and F5) carried monoallelic mutations in the PDX1 gene. Among the fetuses with biallelic PDX1 mutations, fetus F1 had a 2 bp deletion in one allele and a 4 bp insertion in the other allele, whereas fetus F2 had a 30 bp deletion in one allele and a 3 bp insertion in the other allele (Figure 3B).

### Generation of biallelic PDX1-KO piglets by SCNT

PFFs were established from fetus F1, which carried a 2 bp deletion in one allele and a 4 bp insertion in the other allele, and were used as nuclear donors to produce PDX1^−/−^ piglets by SCNT. A total of 462 cloned embryos were transferred to two surrogates in estrus, resulting in the delivery of nine live piglets (Table 3).

**Table 3:** Production of biallelic *PDX1*-KO pigs by SCNT

Surrogate number	No. of embryos transferred	No. of live offspring	Information about the piglets
R115	208	3 (RC1–3)	Diarrhea and vomiting
			All piglets died at day 2
R118	254	6 (RC4–9)	Diarrhea and vomiting
			Two piglets died at day 1
			Four piglets died at day 2

**Figure 4:**
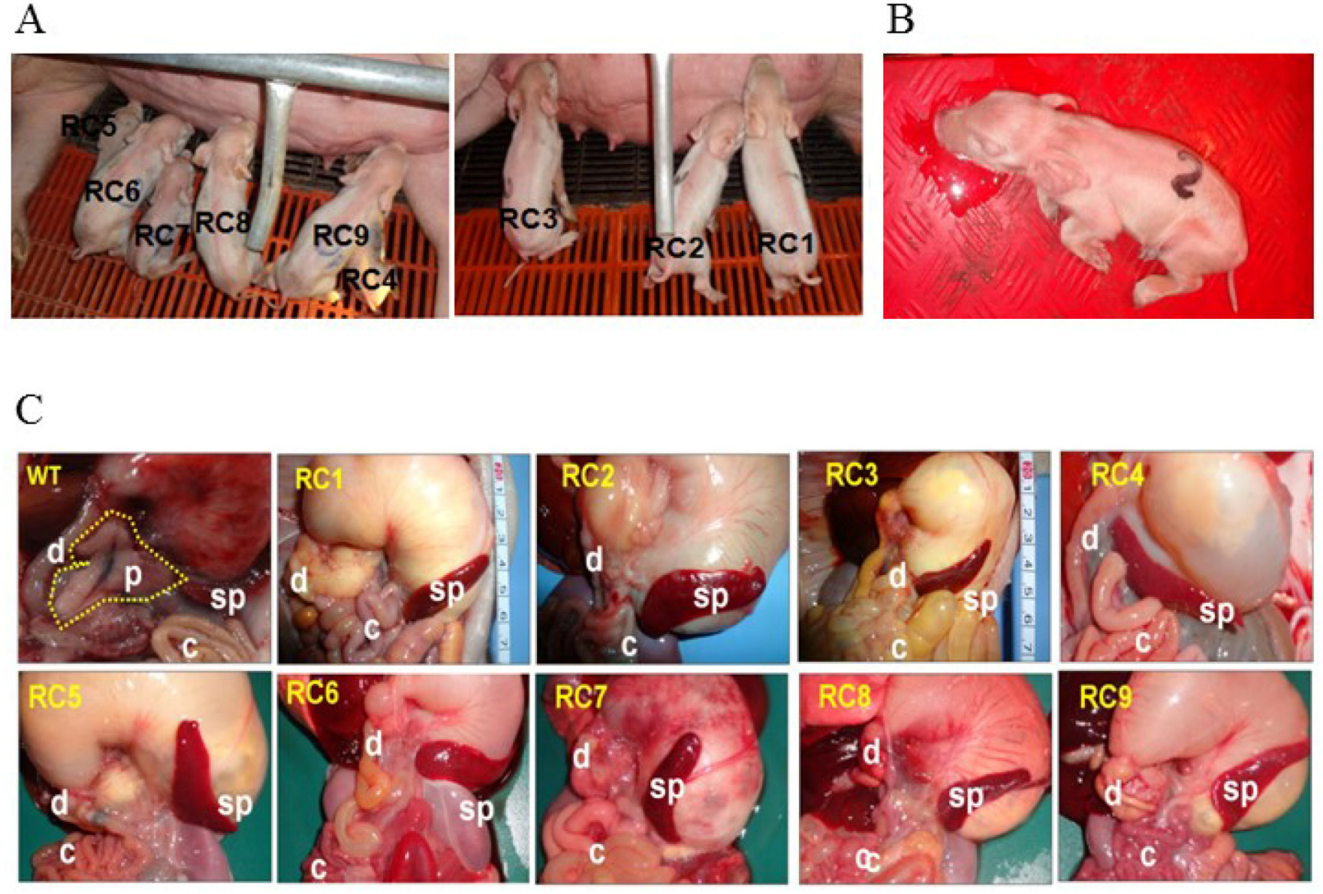
*PDX1*-KO piglets **(A)** Newborn *PDX1*^−/−^ piglets were delivered from two recipients. **(B)** *PDX1*^−/−^ piglets suffered from extreme vomiting before death. **(C)** Analysis of pancreatic development in WT and biallelic mutant pups (RC1–9). A WT piglet had a normal pancreas (dotted line). Faithful reproduction of the pancreatogenesis-disabled phenotype was observed in nine *PDX1*^−/−^ piglets (RC1– 9). s, stomach; p, pancreas; d, duodenum; sp, spleen; c, colon.

**Figure 5:**
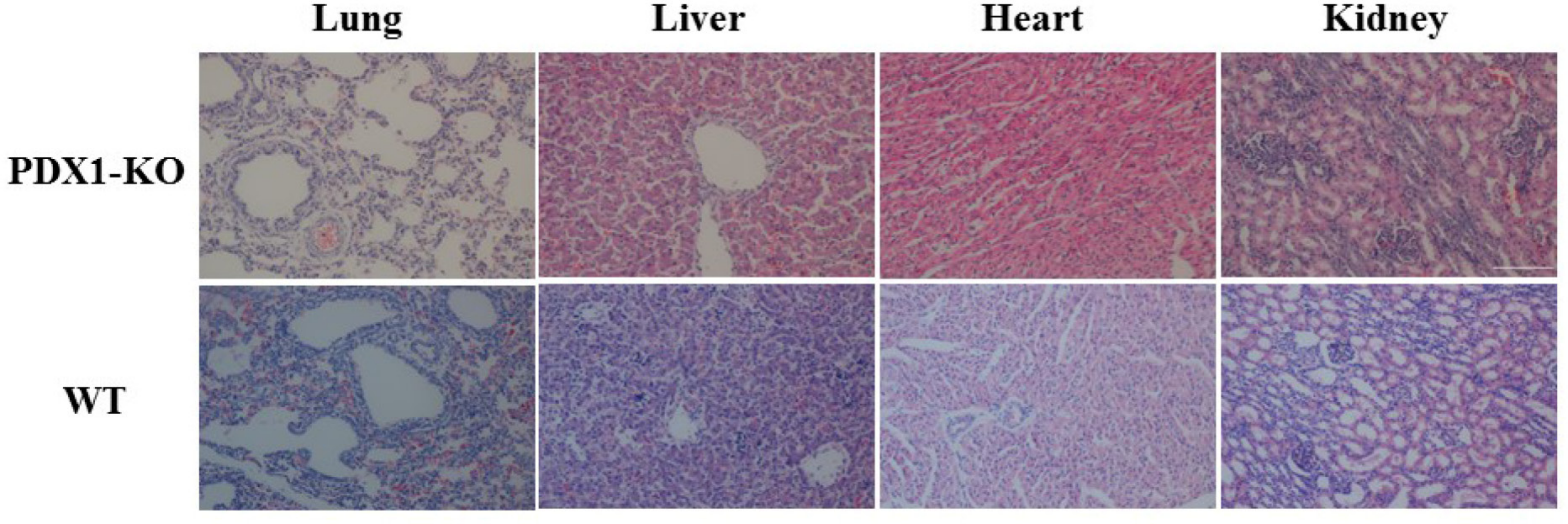
Histological analysis of the main organs from *PDX1*-KO and WT piglets Magnification, 200×. Scale bar indicates 100 μm.

Newborn PDX1^−/−^ piglets were comparable to their WT littermates. However, within the first day postpartum, PDX1^−/−^ animals show signs of growth retardation and dehydration, although they fed because their stomachs contained milk (Figure 4). By 1 day postpartum, the stomachs of some PDX1^−/−^ piglets were distended due to a lack of gastric emptying into the gut (Figure 4C). The serum glucose level was undetectable in these PDX1-null piglets or lower than in WT piglets, and PDX1-null animals showed signs of growth retardation within the first day postpartum (Table 4). These pups often suffered from severe diarrhea and vomiting, and died 2 days after birth. The developmental retardation of PDX1-null pups is likely attributable to malnutrition resulting from a lack of digestion in the absence of the pancreas and functional rostral duodenum and/or diabetic consequences due to the absence of the pancreas. Dissection of PDX1-null pups revealed that the liver, gallbladder, spleen, stomach, common bile duct, and other viscera were present and normal, but the pancreas was noticeably absent (Figure 4). Histological results suggested that the main organs of PDX1^−/−^ piglets displayed no abnormalities (Figure 5).

**Table 4:** Serum glucose concentrations at 1 day after birth and pancreatic phenotype of *PDX1*-KO and WT pigs

Piglet	Blood glucose (mmol/L)^*^	Body weight (kg)		Pancreatogenesis
		1-day-old	2-day-old	
RC1	Low	1.3	1.2 (died)	None
RC2	Low	1.3	1.1 (died)	None
RC3	Low	1.0	0.9 (died)	None
RC4	Low	0.5 (died)	-	None
RC5	Low	1.0	0.9 (died)	None
RC6	2.1	1.0	1.0 (died)	None
RC7	1.7	0.7	0.7 (died)	None
RC8	Low	0.8 (died)	-	None
RC9	1.2	0.9	0.9 (died)	None
WT1	4.6	1.2	1.4	Normal
WT2	3.3	1.1	1.2	Normal
WT3	3.6	1.6	1.7	Normal

^*^Blood glucose levels were measured at 1 day after birth. Low indicates the blood glucose level was lower than 0.6 mmol/L.

## DISCUSSION

One of the cost-effective way is routinely used to generate genetically-modified small animals is directed injection of the gene targeting nucleases into zygote, including rat [15], mice [16], and rabbit [17]. However, many of the resulted founder animals were chimeric ones with multiple mutations through the embryo injection [17]. To get the animals with a single mutation, one or two more round of further breeding have to be employed for selection among the offspring. Clearly, this method is not suitable for large animals, such as pigs, have long gestation cycles, high recipient costs, which make it difficult to achieve large animal offspring with a single expected mutation. SCNT has been successfully applied to generate KO animals in species lacking germline transmitting embryonic stem cells, including pigs, sheep and cattle. In this study, we successfully generated PDX1^−/−^ fetus by specifically targeting the exon 1 site of porcine PDX1 gene with TALEN technology and somatic cell nucleus transfer.

First for SCNT, the fibroblast cell lines were established from one fetus, expanded, and cultured. Cells at an early passage were preserved in liquid nitrogen. Magnetically separated PFFs were used in SCNT, and the results indicated that the competence of reconstructed embryos derived from magnetically separated PFFs did not markedly differ from that of untreated embryos, with a similar rate of blastocyst development and a similar number of cells per blastocyst. Furthermore, pregnancies were maintained by both surrogates implanted with reconstructed embryos derived from magnetically separated PFFs, while other studies reported surrogate pregnancy rates of 36–90% [18–21]. Taken together, our results indicate that surrogate reporter-based cell enrichment is effective for selecting nuclear donor cells with nuclease-mediated gene modification.

To generate PDX1^−/−^ piglets, we used a two-step SCNT method (recloning), in which PFFs derived from the first SCNT were used as nuclear donors for the second SCNT. This recloning method very efficiently generates animals, especially livestock, with targeted gene mutations [22, 23]. Compared with clonally selected transformed fibroblasts, fetal fibroblasts derived from the first SCNT have some advantages for the generation of gene-edited animals. Selection of nuclear donor cells with genetic modifications frequently involves drug treatment of cells in long-term culture. These cultures are inevitably at greater risk of cell exhaustion and senescence [24], which are associated with chromosomal aberrations [25]. Immediately after birth, PDX1^−/−^ piglets were indistinguishable from their WT littermates. However, within the first day postpartum, PDX1^−/−^ animals show signs of growth retardation and dehydration, although they fed because their stomachs contained milk. By 1 day postpartum, the stomachs of some PDX1^−/−^ piglets were distended due to a lack of gastric emptying into the gut. The serum glucose level was undetectable in these PDX1-null piglets or lower than in WT piglets, and PDX1-null animals showed signs of growth retardation within the first day postpartum. These pups often suffered from severe diarrhea and vomiting, and died 2 days after birth. The developmental retardation of PDX1-null pups is likely attributable to malnutrition resulting from a lack of digestion in the absence of the pancreas and functional rostral duodenum and/or diabetic consequences due to the absence of the pancreas [2]. Dissection of PDX1-null pups revealed that the liver, gallbladder, spleen, stomach, common bile duct, and other viscera were present and normal, but the pancreas was noticeably absent, reminiscent of pancreatic agenesis in Pdx1-null mice [2] and human patients [4]. Histological results suggested that the main organs of PDX1^−/−^ piglets displayed no abnormalities.

With unparalleled recent progress in the field of pluripotent stem cells and genome editing, an elegant interspecies chimeric complementation approach is on the horizon to generate functional human organs using animals as hosts [26]. Rat pancreases have been successfully generated in Pdx1^−/−^ mice by combining the principle of blastocyst complementation with the production of interspecific chimeras [27]. A recent report showed that an intermediate type of human induced pluripotent stem cells (hiPSCs) exhibits a higher degree of chimerism and can generate differentiated progenies in post-implantation pig embryos [28]. Therefore, PDX1-KO pigs and hiPSCs can be used to generate human pancreases in pigs via blastocyst complementation, a method that will open up new avenues for organ transplantation [29].

In summary, we used a PDX1-specific TALEN to create frameshift mutations in PFFs, which were subsequently used to generate PDX1 biallelic mutant piglets via SCNT. These piglets at birth were not clearly distinguishable phenotypically from 1-day-old WT piglets, but soon developed severe diarrhea and vomiting and all died within 2 days after birth. Dissection of PDX1-KO piglets revealed that the pancreas was absent in all cases. This would be a useful animal model to study the association of human pancreatic agenesis and diabetes mellitus.

## MATERIALS AND METHODS

### Animals and ethics statement

The protocols of this study were approved by the Committee on the Ethics of Animal Experiments at Yanbian University (Yanji, China). All procedures were performed in strict accordance with the Guide for the Care and Use of Laboratory Animals. All surgical procedures were performed under anesthesia and stringent efforts were made to minimize animal suffering.

### TALEN design and construction

TALEN expression vectors composed of TALEN repeat domains and a FokI nuclease domain containing a Sharkey RR or Sharkey DAS heterodimer mutation for the chosen target sequence were prepared via GoldenGate assembly as described previously [13]. Plasmids harboring the PDX1-targeting TALEN (PDX1-TALEN) were also prepared as described previously [13]. An episomal surrogate reporter containing the chosen target sequence was constructed. TALEN-mediated mutation cells were separated using a column (MACS LS column; Miltenyi Biotech, Germany) according to the manufacturer’s instructions [13]. Plasmids encoding PDX1-TALENs and the surrogate reporter were obtained from ToolGen (Seoul, South Korea).

### Cell culture and transfection

Porcine fetal fibroblasts (PFFs) were established from a fetus at day 32 postconception as previously described [30] and cultured for seven passages in Dulbecco’s modified Eagle medium (DMEM) supplemented with 1% nonessential amino acids, 100 U/mL penicillin, 100 mg/mL streptomycin, and 15% fetal bovine serum (FBS). Cells were nucleofected (Amaxa4D, Lonza) with plasmids at a ratio of 45:45:10 (plasmid encoding a TALEN monomer: plasmid encoding the other TALEN monomer: surrogate reporter). A total of 1 × 10^6^ cells was trypsinized using EDTA-Trypsin (WelGen, Seoul, South Korea), washed with HBSS (WelGen, Seoul, South Korea), centrifuged, and finally suspended in 100 μL supplied buffer containing TALEN and reporter plasmids. Transfected cells were incubated in humidified 95% air with 5% CO_2_ at 37°C for 2d. The expression of fluorescent proteins was assayed 48 h later by flow cytometry. Mutant cells were enriched via magnetic separation as described in a previous study [30]. Fibroblast cells were established from a fetus carrying biallelic mutations and cultured in DMEM supplemented with 15% (v/v) FBS. Cells between passages four and eight were used as donors for nuclear transfer. A single cell suspension was prepared by trypsinization immediately before nuclear transfer.

### Generation of fetuses and offspring by SCNT

Nuclear transfer was performed as described previously [30]. Briefly, mature eggs showing the first polar body were cultured for 1 h in medium supplemented with 0.4 mg/mL demecolcine and 0.05 M sucrose, with sucrose added to enlarge the perivitelline space of the eggs. Treated eggs with a protruding membrane were transferred to medium containing 5 mg/mL cytochalasin B (CB) and 0.4 mg/mL demecolcine, and the protrusions were removed with a beveled pipette. A single donor PFF was injected into the perivitelline space of each egg and the cells were electrically fused using two direct current pulses of 150 V/mm for 50 μs each in 0.28 M mannitol supplemented with 0.1 mM MgSO_4_ and 0.01% polyvinyl alcohol. The fused eggs were cultured in NCSU-37 medium for 1 h before electro-activation. These cells were subsequently cultured in 5 mg/mL CB-supplemented medium for 4 h, followed by activation of the fused eggs by two direct current pulses of 100 V/mm for 20 μs each in 0.28 M mannitol supplemented with 0.1 mM MgSO_4_ and 0.05 mM CaCl_2_. Activated eggs were cultured in medium for 7 days in an atmosphere of 5% CO_2_ and 95% air at 39°C. Cleavage and blastocyst formation were evaluated on Day 2 and 7, respectively. Cloned embryos at the 1-cell stage after fusion or at the 2–4-cell stage after 1 day of culture were transferred into the oviducts of naturally cycling gilts on the first day of standing estrus. Recipient pigs that received the first SCNT embryos were euthanized at day 26–36 of gestation, and the fetuses were collected. These fetuses were used to confirm the PDX1 mutations.

### Detection of mutation and genotyping

T7E1 assays were performed as described previously [31]. Briefly, genomic DNA was isolated using DNeasy Blood & Tissue Kits (Qiagen, Germany), according to the manufacturer’s instructions. The region of DNA containing the TALEN target site was PCR-amplified using the primers listed in Table 5. The amplicons were denatured by heating and annealed to form heteroduplex DNA, which was treated with 5 units of T7E1 (New England Biolabs) for 20 min at 37°C and electrophoresed on agarose gels. To confirm the mutation introduced by TALEN to the target allele, PCR amplicons spanning the target sites were purified using a Gel Extraction Kit (Macherey-Nagel, Germany) and cloned into the T-Blunt vector using the T-Blunt PCR Cloning Kit (SolGent, Daejeon, Korea). The cloned inserts were again amplified using the same primers and sequenced using the M13 primer.

**Table 5:** Primers used in the T7E1 assay

	Direction	Sequence (5′ to 3′)
First	Forward	ctgcgtgcctgtacatgggcc
	Reverse	Gcgtgagccttggtagactt
Second	Forward	ccgccgcctccgttcg
	Reverse	gcgtgagccttggtagactt

### Generation of PDX1^−/−^ piglets and analysis of blood glucose levels

PDX1-KO fetuses were used to generate rejuvenated PFFs for the second round of SCNT to produce boars. Pregnancy was assessed ultrasonographically on day 25. Cloned piglets were delivered naturally or by inducing labor via intramuscular injections of prostaglandin F2α (Ningbo, China) on day 113 of gestation. Blood samples were subsequently obtained by ear marginal veins, and blood glucose levels were measured using a YUYUE™-710 (Jiangsu, China) handheld blood glucose meter.

### Histological analysis

Tissue samples from wild-type (WT) and PDX1^−/−^ pigs were fixed in 10% formalin for 24 h at room temperature and dehydrated in an automated tissue processor. The tissues were embedded in paraffin, sectioned at a thickness of 5 μm, deparaffinized, rehydrated, and stained with hematoxylin for 5 min. The sections were rinsed in running tap water and stained with eosin for 4 min. Hematoxylin and eosin-stained sections were dehydrated and mounted. Images were captured using a Leica DM5000B microscope.

### Statistical analysis

Data are presented as the mean ± SD and are derived from at least three independent experiments. Groups were compared using the Student’s t test, with P values < 0.05 considered statistically significant.

## Abbreviations

PDX1: Pancreatic and duodenal homeobox 1
PDX1-KO: PDX1-knockout
TALEN: Transcription activator-like effector nuclease
SCNT: Somatic cell nuclear transfer
PFFs: Porcine fetal fibroblasts
WT: Wild-type
